# Visualizing dementia and stigma: a scoping review of the literature

**DOI:** 10.1177/14703572241245587

**Published:** 2024-05-18

**Authors:** Emma Putland, Gavin Brookes

**Affiliations:** Lancaster University, UK

**Keywords:** dementia, discourse, images, scoping review, stigma, visual

## Abstract

Discursive choices are recognized by both academic and dementia communities as being central to the perpetuation (or challenging) of dementia-related stigma. Yet, the focus of the vast majority of research on the discursive dynamics of dementia stigma to date has been on the role of language only, effectively failing to regard the multimodal reality of discourse. The present study aims to address this gap by conducting a scoping review of the smaller, and relatively more recent, body of literature that has examined visual modes of communication. The authors ask the following questions: (1) What theories of stigma have informed or guided studies of visual representations of dementia and people with dementia? (2) What visual features of representations of dementia and people with dementia might contribute to and/or challenge dementia stigma? Using Scopus, PubMed, PsychInfo and Google Scholar, 10 papers published between January 2000 and July 2023 were selected and thematically synthesized. The authors found that most studies had limited or no engagement with specific stigma theories, although the general principle of establishing or challenging distance between an in-group (‘us’) and out-group (‘them’) informed many of the analyses. Visual features with the potential to contribute to stigma tended to impersonalize people with dementia through foregrounding visual markers of dementia (oftentimes emphasizing loss and/or the brain) and establishing symbolic distance between viewers/other represented participants and people with dementia. This distance could be achieved through visual framing techniques (regarding angle, gaze, colour, setting) which, for instance, could subtly position people with dementia as the ‘living dead’. There was much less focus on visual features with the potential to challenge stigma, which together emphasized social connection, transformation and taking the perspective of someone with dementia. Turning to reception, another potential aspect of challenging stigma was reinterpreting supposedly ‘stigmatizing’ images. In this article, these findings are interpreted in relation to the broader stigma literature and implications for future research and advocacy efforts are discussed.

## Introduction

Dementia stands as one of the most pressing public health issues of our era, affecting 50 million individuals worldwide. Moreover, this figure is on an upward trajectory, with an estimated 7.7 million new cases emerging each year. As such, it is estimated that the number of people currently living with a dementia diagnosis will have tripled by the year 2050 (Alzheimer’s Research UK, 2018). Understanding dementia is a complex undertaking, not merely from a medical perspective but also within broader social and cultural contexts. The concept of dementia is a ‘slippery’ one, as perceptions of it are heavily influenced by current societal views that commonly associate the syndrome with the fears surrounding ageing, loss and mortality (Zeilig, 2014: 260). Clinically, dementia is an umbrella term that encompasses various disease processes, all of which result in cognitive impairment. Symptoms typically include memory loss, as well as difficulties in reasoning, perception and communication.

Since there is presently no known cure for dementia, it remains a chronic condition. Consequently, those diagnosed with dementia face a lifetime of challenges associated with the syndrome. Yet people diagnosed with dementia must live not only with the health challenges that the syndrome presents, but also with the stigma that surrounds it. In his seminal work on the topic, the sociologist Erving Goffman (1963) defined *stigma* broadly as a ‘spoiled identity’ in which the stigmatized aspects of a person’s identity are often used to define them. The stigma that surrounds dementia – often referred to as ‘dementia stigma’ – has been observed to be a salient feature of the experience of living with the syndrome (Swaffer, 2014). Yet, beyond this rather broad label, a number of different types of stigma have been identified across the vast literature on this topic, including in relation to dementia specifically. This includes, but is not limited to, stigma amongst the general public, self-stigma, stigma through association with the stigmatized person, provider-based stigma and structural stigma (e.g. Link and Phelan, 2001; Nguyen and Li, 2020; Pescosolido and Martin, 2015; Werner, 2014).

Dementia stigma can have far-reaching consequences that impact not only those living with the syndrome but, indeed, society at large. For people diagnosed with dementia as well as their families, dementia stigma can engender feelings of shame, thus discouraging individuals from seeking timely medical intervention or participating in research (Swaffer, 2014). This stigma has also been found to adversely influence the design and delivery of healthcare for those individuals, from funding decisions to service commissioning and frontline care (Benbow and Jolley, 2012). In these respects, dementia stigma can adversely impact not only the quality of life of people with dementia, but indeed their life chances too.

In wider society, dementia stigma has been argued to contribute towards negative, and even inaccurate, public perceptions of the syndrome. Research from Alzheimer’s Research UK indicates that misunderstandings about dementia are prevalent; for example, one in five UK adults incorrectly believes that dementia is an inevitable part of aging. Such misconceptions contribute to negative attitudes and make meaningful communication with people with dementia more challenging for the general public (Alzheimer’s Research UK, 2018). A report by Alzheimer’s Disease International further highlights that as many as 80 percent of people are fearful of dementia, with many being fearful of interacting with people with dementia (Alzheimer’s Disease International, 2019). Such attitudes, in turn, can lead to increased feelings of shame in people with dementia and contribute further to their social isolation. Indeed, as Kate Swaffer, an advocate living with dementia, put it, ‘the ugliest part of having dementia is probably the reaction of others’ (Swaffer, 2016: 66).

The collective harm caused by dementia stigma is profound then, influencing not just the health and well-being of those directly affected but also shaping societal attitudes and healthcare systems. This has implications for relatives and caregivers and, ultimately, affects society as a whole (Alzheimer’s Disease International, 2019). Challenging such stigma could therefore yield multiple benefits, including more timely diagnoses, improved quality of care, improved attitudes towards people with dementia amongst the general public and a reduction in societal misunderstandings about the syndrome. One of the primary means through which processes of stigmatization, with respect to dementia but other phenomena too, can be understood and challenged is through the discourses that we, as a society, use to communicate about the syndrome.

Discourses, here, can be viewed as consistent linguistic and visual practices that construct partial representations of reality, with different communicative choices establishing different ways of viewing the world and objects, people and events in it (Mills, 2005), including, for our purposes, dementia and people with dementia. Indeed, an appreciable and growing body of work indicates that the stigmatizing ideologies that surround dementia can be both perpetuated by, but also contested through, what we might broadly term ‘discourse’ (e.g. Brookes, 2003; Putland, 2022; Swaffer, 2014). Such work has demonstrated how public discourse around dementia tends to be negative and sensationalistic, emphasizing the threat posed by dementia, depicting those diagnosed with it as hazardous and diminished versions of their past selves, and ultimately going so far as to equate dementia diagnosis to a death sentence. Such prominent portrayals not only perpetuate harmful stereotypes but, importantly, contribute to dementia stigma, and thus have the potential to engender feelings of shame among those diagnosed with dementia and their families, as well as fear and misunderstanding among the public (Burgener and Berger, 2008). Accordingly, academics, advocacy groups and dementia charities alike have emphasized the need for a more nuanced and respectful public discourse (Swaffer, 2014). The need to continually scrutinize and modify such representations, then, constitutes not just an academic concern but a social imperative.

Responding to this need, charities and advocacy groups have developed numerous sets of guidelines to help those communicating about dementia to do so in a way which, broadly, seeks to reduce stigma and instead promote a more person-centred view of those diagnosed with dementia, as well as a more genuine awareness of the syndrome (e.g. Alzheimer Europe and European Working Group of People with Dementia, 2022; Alzheimer’s Society, 2018; Bould, 2018; DEEP, 2014). Likewise, and broadly in aid of such initiatives, a growing body of academic research has begun to address the discursive mechanisms through which dementia stigma is propagated (Putland and Brookes, 2024). Notably, such attempts at understanding and challenging dementia stigma in discourse have focused, in the main, on the role of language use in this regard. However, prominent sites of public discourse around health and illness topics, including dementia, are characteristically multimodal (Brookes et al. 2021), often incorporating language alongside other semiotic modes, such as visual communication, which is the focus of this scoping review. As well as being highly pervasive, visual modes in particular are arguably able to more subtly naturalize and reproduce certain versions of reality, in the process providing us with ‘views of how things should be’ (Harrison, 2002: 857).

Moreover, visual practices are central to cultural understandings of difference, and to the stigmatization of these (Kessler, 2022). People are evaluated according to culturally constructed categories (such as normal/abnormal, attractive/repulsive, able/disabled and superior/inferior) and certain bodies are subsequently delegitimized relative to others (Garland Thomson, 1997). Such a process requires recognizable markers of difference, which contributes to pressure for stigmatized individuals to at times conceal, and at other times, reveal visual markers of difference (Kessler, 2022). For a neurodegenerative and diverse syndrome such as dementia, visual markers may be less physically clear, and thus the representative choices made require further scrutiny.

In this article, we seek to consolidate existing insights into the visual discourses through which dementia stigma is created but also challenged. Specifically, we conduct a scoping review of the limited research that has addressed the visual dynamics of stigmatizing discourses around dementia. While similar to systematic reviews in the sense that they follow a structured process, scoping reviews tend to have a broader focus, aiming to map out the research literature for a particular topic, irrespective of evaluating study quality, which makes them useful for examining emerging areas, clarifying concepts, identifying key characteristics relating to a concept and observing research gaps (Munn et al., 2018; Tricco et al., 2016). Here, we take a thematic approach to research synthesis (see Popay et al., 2006; Thomas and Harden, 2008; Tong et al., 2012), in order to answer the following questions: (1) What theories of stigma have informed or guided studies of visual representations of dementia and people with dementia? and (2) What visual features of representations of dementia and people with dementia might contribute to and/or challenge dementia stigma? These research questions also drive our corresponding scoping review of language and dementia stigma (Putland and Brookes, 2024).

Our first research question responds to previous observations that the concept of ‘stigma’ is overused but underdefined in the social sciences (Nguyen and Li, 2020: 149) by aiming to chart if and how studies of visual communication theorize stigma, and to consider the extent to which this informs the interpretation of visual communication. Considering the impact of dementia stigma discussed above, a more in-depth exploration of this concept has the potential to usefully inform both the interpretation and production of visual texts.

Through our second research question, we aim to bring together otherwise disparate insights into the visual means by which dementia stigma can be created and challenged. Synthesizing existing research in this way has the potential to shed new light on broader patterns, consistencies and discrepancies in the (visual) discursive dynamics of dementia stigma. This more holistic perspective – taking in, as it does, various genres and contexts of communication – is, in our view, particularly beneficial regarding visual analyses. This is because such studies are usually more suited to qualitative as opposed to quantitative methods of analysis, and so each focuses (often necessarily, and to great benefit in terms of analytic granularity) on a single genre or communicative context. The insights provided through this review are therefore intended to support future initiatives that help develop communicative practices that challenge and reduce, rather than propagate, dementia stigma (e.g. communication guidelines and training – which, as noted, focus primarily on linguistic rather than visual communicative choices). For those intent on interrogating visual representations of dementia and people with dementia in the future, this review highlights current gaps to be addressed in future research, including how theorizations of stigma can enrich such analyses.

## Methodology

### Screening studies

The flowchart in Figure 1 visualizes our process for searching and filtering results. This was done in two stages to enable an up-to-date review of the literature following our initial scoping review of language, dementia and stigma (see Putland and Brookes, 2024). Through pilot testing using Scopus, we selected the search terms shown in Figure 1, which were devised to maximize the heterogeneity of the results, thus spanning various disciplines and theoretical models of communication. No alternative terms were offered for ‘stigma*’ since we wanted to study engagement with this concept specifically. While the search terms incorporate a multimodal perspective on discourse, due to space limitations, this review focuses only on *visual* representations of dementia.

**Figure 1.**
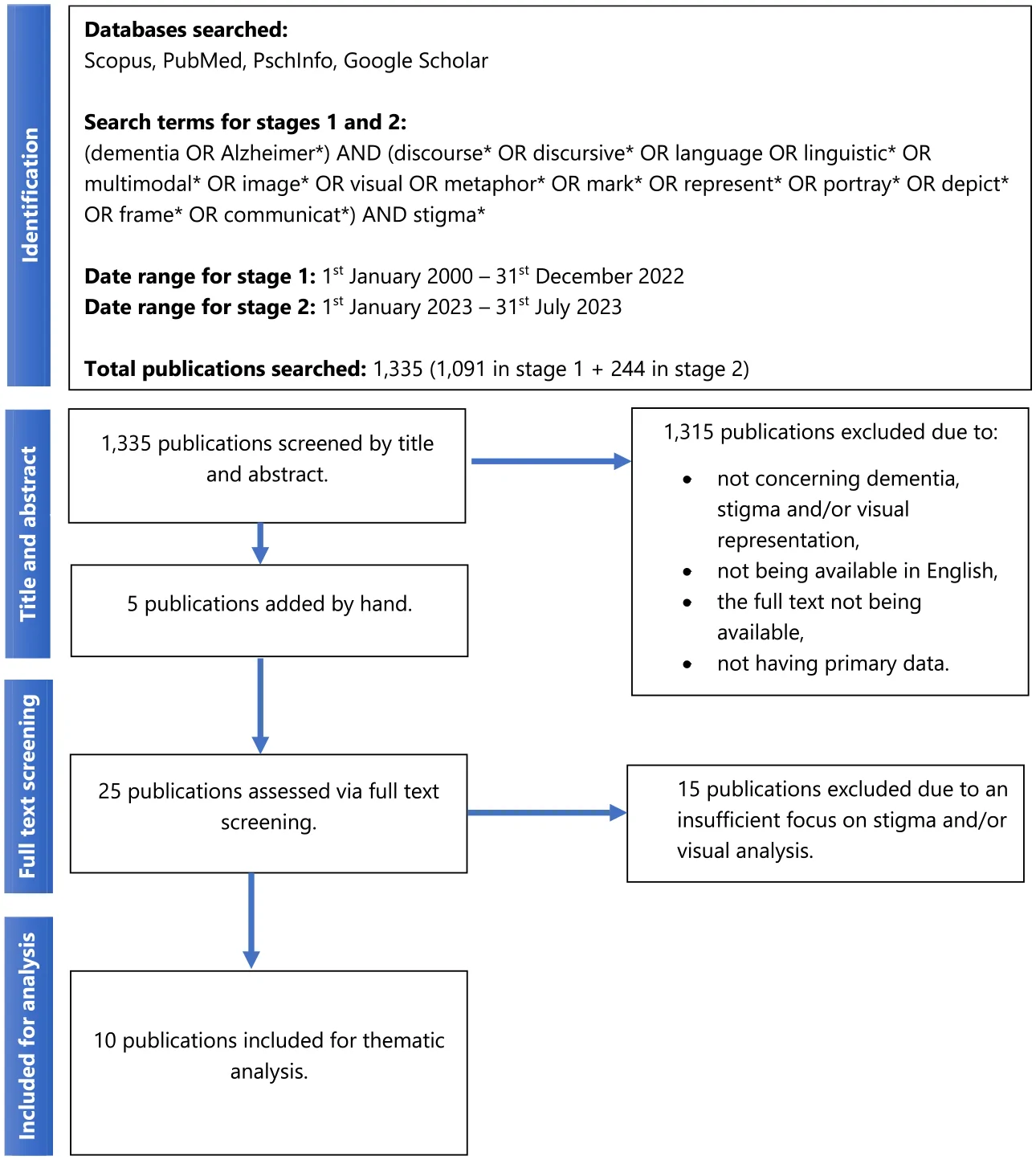
A summary of the screening process.

The electronic databases Scopus, PubMed and PsychInfo were selected to cover the social sciences, arts and humanities, and health. Titles, abstracts and (where possible) keywords were searched in January 2023 for publications dated between 2000 and 2022 inclusive (stage 1 search), and again on 1 August 2023 to find any additional publications released between January and July 2023 inclusive (stage 2 search). The same two-stage search was conducted using the web-based search engine, Google Scholar; we used the search engine’s facility to automatically sort the results by ‘relevance’ and screened the first 250 results during the stage 1 search and the first 150 results during the stage 2 search, which were both points at which relevant results had not appeared for approximately the last 50 results shown. A further five publications were added by hand for full-text screening following a supplementary manual search of relevant reference lists and by drawing on our prior knowledge of the field.

For inclusion in this scoping review, we determined that publications must meet all of the following criteria:

- Contain original research on visual representation of dementia;
- Contextualize some or all findings in relation to dementia stigma;^1^
- Have been published between 1 January 2000 and 31 July 2023 (inclusive);
- Include analysis of primary data (i.e. original empirical data);
- If concerning audiovisual data, must provide analysis of at least one still image and not rely solely on other aspects of a video/film for interpretation;
- Can access the full publication;
- Be written in English with either English language data or English translations of data in other languages.

Publications for full text screening were sorted in the referencing manager, Zotero. Eligible publications were read independently by both authors, who then resolved borderline cases for inclusion through discussion. Nine articles and one book chapter were included in the subsequent review; Table 1 summarizes the research aim/questions, data, cultural contexts and analytical approaches identified for each of these publications. While, in conducting and reporting our review we have referred to Tong et al.’s (2012) guidance for enhancing transparency in reporting the synthesis of qualitative research, we have maintained a critical and reflexive approach to our review, rather than simply adhering to a checklist. Notably, quality rating tools were deemed inappropriate for our review of these qualitative studies, and thus no evaluative component was included in this scoping review (see Morse, 2022).

**Table 1.** Characteristics of the publications in the review.

Authors, date	Title	Research aim/questions	Data analysed	Cultural context under study	Analytical approach
Ang et al. (2023)	Advocating for a dementia-inclusive visual communication	To examine underlying visual communication strategies of images of dementia in the public domain, with the aim of informing and advocating for a dementia-inclusive visual communication.	432 images from a Malaysian mainstream English newspaper (2012–2021), the Alzheimer’s Disease Foundation Malaysia website (2019–2021) and the Ministry of Health Malaysia’s website (2019–2021).	Malaysia	Social semiotics, specifically the Visual Discourses of Disability (ViDD) framework. Consulted four representatives of Alzheimer’s Disease Foundation Malaysia to corroborate findings and verify the plausibility of proposed recommendations.
Brookes et al. (2018)	‘Our biggest killer’: Multimodal discourse representations of dementia in the British press	To critically examine how the British press reported the findings of the 2016 Office for National Statistics’ bulletin that dementia is ‘the leading cause of death’ in England and Wales, particularly attending to the recurring discursive–semiotic strategies used to frame dementia as a dreaded and devastating disease.	11 articles by 10 national newspapers in the UK published on 14–15 November 2016.	UK	Multimodal critical discourse analysis
Brookes et al. (2021)	Multimodality: Examining visual representations of dementia in public health discourse	To examine how dementia is constituted through discourse, including through both linguistic and non-linguistic means, in an awareness-raising campaign.	Three parallel posters produced as part of an awareness-raising campaign by the UK charity, Alzheimer’s Society and the National Health Service (NHS).	UK	Multimodal critical discourse analysis
Caldwell et al. (2021)	Depicting dementia: Representations of cognitive health and illness in 10 picturebooks for children	To examine what picturebooks for young children communicate about dementia, both visually and textually.	The images and text in 10 randomly selected picturebooks about dementia (from a corpus of 46). Published between 2002–2017.	UK, Australia, New Zealand, USA, Canada, Belgium	A close visual and textual critical discourse analysis that adapted Caldwell and Wilbraham’s (2018) methodology for qualitative visual analysis.
Harvey and Brookes (2019)	Looking through dementia: What do commercial stock images tell us about aging and cognitive decline?	To critically examine the visual discourses surrounding dementia in a large sample of stock images.	The most popular 100 images from a search for ‘dementia’ in the Getty image bank.	American company	Multimodal critical discourse analysis
Kovan and Soled (2023)	A disembodied dementia: Graphic medicine and illness narratives	To present embodied selfhood as an alternative anthropological framework (to the view of neurological decline as a loss of self). To propose graphic medicine as a tool through which this theory can be applied to caregiving practice. To demonstrate tensions in conceptions of selfhood evident across three different graphic novels related to dementia.	Three graphic novels (*Wrinkles, Tangles* and *Aliceheimer’s*).	France, Spain, Canada, USA	Social constructionist approach to dementia, focusing on embodied selfhood.
Latimer (2018)	Repelling neoliberal world-making? How the ageing–dementia relation is reassembling the social	To examine the social and existential significance of an emergent biopolitics around the ageing–dementia relation and argue that this politics represents a shift from emic to phagic strategies for incorporating, assimilating and transforming the Otherness that the ageing–dementia relation is made to represent.	Film/video, poetry and infographics.	UK and USA oriented	Applying Bauman’s theories of coping with Otherness and theories of the rationale for the making of monsters to examine the ageing–dementia relation.
Venkatesan and Ancy (2023)	Care as a creative practice: comics, dementia and graphic medicine	To demonstrate, through close analysis, how care as creative practice provides a therapeutic direction when biomedical cure becomes impossible.	Dana Walrath’s (2016) graphic memoir, *Aliceheimer’s: Alzheimer’s Through the Looking Glass*	USA	Not specified
Venkatesan and Kasthuri (2018)	‘Magic and laughter’: Graphic medicine, recasting Alzheimer narratives and Dana Walrath’s *Aliceheimer’s: Alzheimer’s Through the Looking Glass*.	To examine how Walrath’s *Aliceheimer’s* cultivates alternatives to the biomedical and cultural figurations of AD through the use of collage form, positive lexical choices and a creative appropriation of *Wonderland*.	Dana Walrath’s (2016) graphic memoir, *Aliceheimer’s: Alzheimer’s Through the Looking Glass*	USA	Not specified
Vermeer et al. (2022)	Selling surveillance technology: Semiotic themes in advertisements for ageing in place with dementia	To examine the interplay between surveillance technology advertisements and audiences, and to reflect on what it means for this discourse and market.	Six websites with video and print advertisements for surveillance technology. Two focus groups of four carers and of five people living with dementia who responded to the advertisements.	The UK, Sweden, and the Netherlands.	A tripartite approach to media literacy (production, semiotic textual analysis and audience reception). Data analysed using qualitative content analysis and a critical discourse approach to semiotic analysis of the adverts.

### Analytical approach

Descriptive analysis of the studies’ characteristics (data types, approaches, etc.) were conducted and recorded using tables for each characteristic of interest. Uses of the concept of stigma were recorded through a combination of close reading and searching for every use of the word ‘stigma’ and its grammatical variants (e.g. ‘stigmatizing/stigmatising’). For identifying visual features that might contribute to and/or challenge dementia stigma, we followed Thomas and Harden’s (2008) approach to thematic synthesis. First, relevant findings and arguments relating to stigma and associated visual representations were manually coded line-by-line, from which descriptive themes were generated by each author and collaboratively revised as we progressed through the studies. Although many findings were explicitly linked to stigma by the publications’ authors, some required more interpretation on our part, at which point we considered the publication’s overall framing of its results and whether the findings fitted into the descriptive themes that we had identified. Broader analytical themes were then developed collaboratively to reflect how visual features might contribute to and/or challenge dementia stigma. While theme generation was inductive, it was inevitably influenced by our prior knowledge of dementia, stigma and visual communication research. The results of our analysis are presented in the following section, which begins by summarizing the publications’ overall characteristics before considering how stigma is theorized across the studies, and the visual features that are identified as contributing to and/or challenging dementia stigma.

## Results

### Study characteristics

It is useful to begin with an overview of the included studies’ characteristics (see Table 1) in order to consider both patterns and potential gaps in the existing literature. Notably, the date range indicates that interest in visual representations of dementia and stigma is a recent phenomenon; no papers were published between 2000–2017, with four being published between 2018 and 2020 and six between 2021 and 2023. These studies featured in a range of primarily social science and humanities contexts, including social semiotics, health/medical humanities, dementia and literature journals. None of the authors of the studies reviewed were reported to have dementia or be a care giver.

Methodologically, the studies are overwhelmingly qualitative and draw on a range of data types. The main data types combine text with images, namely books (both graphic novels and children’s picturebooks), newspapers, website pages, infographics, public health poster campaigns and visual print advertisements. Two studies include an analysis of videos, and two complement their visual analysis with the responses of participants in focus groups and interviews, which include four representatives of a national charity (Ang et al., 2023), and five people living with dementia and four carers (Vermeer et al., 2022). The included data types have a range of purposes, notably for entertainment, service/product promotion and education.

Regarding analytical approaches, what we might broadly refer to as Multimodal Critical Discourse Analysis (MCDA) is particularly popular; this is explicitly discussed by three studies and referred to using another name by two more (i.e. visual and textual CDA and a critical discourse approach to semiotic analysis), while a sixth study by Ang et al. (2023) aligns itself with theories and aims that are formative to MCDA, establishing an implicit association. Essentially, MCDA is concerned with how macro social issues (e.g. dementia) are expressed and enacted at the micro level of social texts, and in critically analysing these examples in relation to existing social structures (Van Dijk, 2009). Most of these studies seem to draw on the social semiotic tradition, which is concerned with signs (that consist of the *signifier*, forms that a sign can take and the *signified*, the mental concept behind the sign) and sign-using behaviour. In other words, the studies regard particular semiotic choices (e.g. regarding facial expressions, angles and colour) as ideologically laden and able to impact how dementia and people with dementia are conceptualized and positioned in society. Three of the studies presuppose a visual grammar, drawing on Kress and Van Leeuwen’s (2002, 2006) categorizations of visual composition (Brookes et al., 2021; Harvey and Brookes, 2019) and Van Leeuwen’s (2008) visual social actor and viewer frameworks (Ang et al., 2023). From this, Ang et al.’s (2023) study proposes a specific framework, Visual Discourses of Disability, which orients around two clines of visual representation: perspectivizing–personalizing and disabling–enabling. Additionally, Ang et al. (2023) and Vermeer et al. (2022) conduct qualitative content analysis of interviews and focus groups to consider production and audience reception alongside the authors’ analysis of the social texts. Four studies do not clearly specify an analytical approach, although two clearly signpost theories that inform their analysis, namely regarding the social construction of dementia, selfhood theories, coping with Otherness and making monsters (Kovan and Soled, 2023; Latimer, 2018).

Likely reflecting our search criteria, this dataset shows a clear bias towards the English language and Western contexts. All studies draw on English language data, although two studies imply the presence of other languages, namely Swedish and Dutch (Vermeer et al., 2022) and Malay (Ang et al., 2023). The UK and USA are the most popular cultural contexts for studies (featuring in five and six studies, respectively), with other European (France, Spain, Sweden, the Netherlands and Belgium), North American (Canada) and Oceanian (New Zealand) countries also being featured. Only one study looks beyond this Western context, with Ang et al. (2023) analysing Malaysian media and organisations.

### Theorizing stigma

Although all of the publications mention stigma, this concept seems to have more of an implicit influence on the analytical approaches taken, rather than guiding or informing the approaches taken in any explicitly marked way. Indeed, in only three of the ten papers do the authors actually define what they understand the term ‘stigma’ to mean. That 70 percent of included papers do not seek to theoretically outline stigma aligns with Werner’s (2014) review of dementia stigma and adds further support to Nguyen and Li’s (2020: 149) observation that, while the concept of stigma is ‘overused’ in the social sciences, it typically remains ‘underdefined’ or is ‘not explicitly defined’.

When stigma is defined by the studies surveyed, the authors do so in reference to Goffman’s (1963) seminal work on the topic. Papers either focus on stigma as a discrediting *attribute* that therefore ‘marks’ a person with a stigma (Ang et al., 2023; Brookes et al., 2018) or as a *relation*, whereby someone is marginalized when they do not fit the world of others and have difficulty passing as ‘one of us’ (Latimer, 2018: 833). Of course, as Brookes et al. (2018) acknowledge, both approaches are interlinked, since any discrediting attribute *is* relational, in the sense that any attribute’s status, for example as discrediting, is determined by societal (i.e. social and thereby necessarily relational) ideologies. While Goffman’s work is seminal for stigma, it is notable that no other theorizations of the concept are drawn upon by these publications (e.g. see Corrigan et al., 2005; Jones et al., 1984; Link and Phelan, 2001; Pescosolido and Martin, 2015; Smith, 2007). Nor are there any references to theorizations around the challenging of stigma (e.g. Bacsu et al., 2022; Clair et al., 2016; Corrigan and Penn, 1999). Having said this, authors do commonly explore a central aspect of stigma theories in general – that of establishing (or challenging) distance between an in-group (‘us’) and out-group (‘them’). This is a point we shall return to shortly.

Although stigma is not widely defined within the research under review, the concept is nevertheless discussed in a variety of contexts which position it broadly as what Latimer (2018: 946) refers to as ‘the effect and affects of complex alignments: political, medical [and] cultural’. Notably, the intersectionality of dementia stigma is observed, particularly in relation to ageism, in that ‘ageing when it is associated with dementia is doubly stigmatizing’ (p. 833). Different aspects of dementia (namely, symptoms being perceived as abnormal; Harvey and Brookes, 2019) and wider social discourses that influence notions of personhood (i.e. neoliberalism, loss-of-self; Kovan and Soled, 2023; Latimer, 2018) are postulated as contributors to dementia stigma. Yet stigma is dynamic as a concept, as highlighted by Latimer's (2018: 832) discussion of a neoliberal-inspired ‘new stigma’, which goes beyond ‘simply having dementia’ and becomes ‘failing to prevent it’. While stigma is not comprehensively theorized by these studies, then, authors do clearly draw on aspects of stigma theories and, as we will demonstrate, present numerous relevant arguments regarding how stigma can be discursively constructed and/or challenged, here specifically focusing on how this might be achieved through visual representations.

### Contributing to dementia stigma?

While numerous visual features with the potential to contribute to stigma can be identified across the included studies, the underpinning strategy appears to be that of distancing viewers from represented participants with dementia. Below, we outline how such distance is evoked in more detail, which can broadly be divided into focusing on dementia above the person, and emphasizing differences between ‘us’ (the viewers and/or people without dementia) and ‘them’ (the represented participants who appear to have dementia) through both the attributed characteristics and physical positioning of those represented participants. Of course, in reality, representing dementia *above* the person and representing *the person* are inextricably interlinked, and therefore it is important to highlight that the below categories are presented here simply for the purposes of clarity.

#### Representing dementia above the person

Many of the studies observe that images that include people with dementia (in either a photorealistic or an abstract sense) seem to focus on dementia and its symptoms above the individual experiencing the syndrome. For instance, images might show people with their hand on their forehead (or otherwise pointing to their brain) with a pained facial expression, which may direct viewers to look at manifestations of dementia – here, at suffering and/or confusion – ‘before and perhaps instead of the person’ (Ang et al., 2023; Brookes et al., 2018: 384). Confusion, memory loss and disengagement can also be conveyed through vacant, emotionless expressions (e.g. Brookes et al., 2021) or, conversely, through lost and fearful ones (Venkatesan and Kasthuri, 2018). People with dementia are either shown in passive, immobile positions (a point that we shall return to) or as otherwise vulnerable, through being post-fall or getting lost (Ang et al., 2023; Venkatesan and Kasthuri, 2018). In chronological narratives, ‘before’ and ‘after’ images can be used to signal a represented participant’s dementia, which usually entails the represented participant appearing frailer, physically less able, older (e.g. through more wrinkles) and increasingly ‘stagnant’ (Caldwell et al., 2021; Kovan and Soled, 2023: 235). Frequently, images convey the act and consequences of forgetting, which range from showing incomplete hobbies (such as crochet) or unusual behaviour (e.g. putting shoes in the fridge) in children’s picturebooks (Caldwell et al., 2021), to potentially dangerous scenarios, such as leaving the car door open, leaving hot appliances turned on, or forgetting about a running tap (Brookes et al., 2021; Kovan and Soled, 2023). In the context of an infographic, people with dementia are implicitly associated with danger not through their symptoms but through their numbers and the economic cost they pose to society (Latimer, 2018). These are all examples of abstraction (namely, representing people through the qualities associated with dementia; Ang et al., 2023; Van Leeuwen, 2008), which risks impersonalizing the individual through instead using them to foreground markers of dementia, here memory loss, danger and suffering.

People living with dementia can also be impersonalized through various forms of objectivization. A prominent visual trope is that of using extreme close-up shots to show certain body parts, which in the context of dementia representation are overwhelmingly fragile hands or damaged brains (Ang et al., 2023; Brookes et al., 2018; Harvey and Brookes, 2019). Showing only a hand or brain is a visual form of somatization (representing someone via their body part; Van Leeuwen, 2008: 47), which objectifies and reduces a person with dementia to a specific anatomical feature that is removed from their whole body, and thus their self. Such disembodiment therefore depersonalizes the represented participant, particularly by excluding their face, which is popularly assumed to be the key conveyor of personality and identity (Archer et al., 1983; Harvey and Brookes, 2019).

Close-ups of hands and brains have distinct connotations in a dementia context, although each tends to perpetuate the discursive association of dementia with degeneration. Firstly, shots of hands overwhelmingly depict the hands of older adults, likely aged 70+. These hands are often brightly lit, which, combined with the detail afforded by a close-up shot, emphasizes visual markers of ageing such as wrinkles and bones, which in turn can connote fragility, bodily decline and conflate dementia with older age (Ang et al., 2023; Brookes et al., 2018). This emphasis on older age is notable since the ageing body is often positioned as the site of difference and disgust (Latimer, 2018; Van Wijngaarden et al., 2019). If images only show the hands of one person, these tend to either rest passively or be clasped tightly as if in pain, arguably evoking both vulnerability and isolation (Ang et al., 2023; Brookes et al., 2018). Other close-up hand shots interact either with the hand of a carer or a medical device (e.g. a wheelchair, medical bracelet or walking aid), which can again suggest vulnerability and dependence, although potentially also support (Ang et al., 2023). Consistently, then, the hands of older adults are framed in ways that can connote vulnerability and distress, especially when depicted alone. However, a direct association of the hand trope with perpetuating stigma is complicated by the potential of hands interacting to instead model more inclusive and supportive positionings of people with dementia, which will be explored further in relation to challenging stigma.

The brain is a visual trope that we shall return to frequently since it appears to be the body part whose visual representation has been observed most often as having stigmatizing potential in a dementia context. This is perhaps unsurprising, considering the neurodegenerative nature of dementia, and the social importance assigned to an efficiently functioning brain which, in contemporary ‘hypercognitive’ societies, is regarded as the locus of human identity and worth (Post, 2000a, 2000b). Sometimes, neuroimaging scans of the brains of people with dementia can be used in place of images of the person themselves. Neurological images hold ‘immense rhetorical and persuasive power’ (Brookes et al., 2018: 387) since they are widely associated with neuroscientific expertise and objectivity, and the promise to ‘show’ not only the interior of humans, but the physicality of dementia as a syndrome (Dumit, 1999, 2004). As such, brain scan images can be used to evoke reliability, to legitimize information and to convince readers of the prevalence of dementia (Ang et al., 2023). Yet, without a comprehensive explanation in the contexts of their use, the medical implications of these authoritative images are likely to be somewhat incomprehensible to non-expert viewers. Subsequently, such images risk being interpreted as showing a diseased (as opposed to a healthy) brain. Considering the importance afforded to the brain for marking identity and personhood – or what Vidal (2009) appropriately terms, ‘brainhood’ – a diseased brain may even be extended to represent a ‘diseased’ person, contributing to constructing a healthy/normal ‘us’ versus a diseased/abnormal ‘them’ (Brookes et al., 2018; Harvey and Brookes, 2019).

Sometimes the head and/or face of a represented participant with dementia is included but is importantly *backgrounded* in favour of displaying the person’s (faulty) brain. This is especially the case with loss-oriented visual metaphors, which are observed within half of the studies we reviewed. Overwhelmingly, people living with dementia are depicted as having disintegrating brains, which entails both the somatization and abstraction of people with dementia, who are represented through their impaired body part, namely the brain (somatization) and through visualizing symptoms and other aspects of dementia, such as neurodegeneration (abstraction) (Ang et al., 2023). Research indicates that such visual metaphors can be interpreted as meaningful explanations of otherwise complex neurological processes, yet they also have the potential to reproduce stigmatizing discourses of loss of self and hopelessness with dementia (Ang et al., 2023; Putland, 2022). Many of the metaphorical source domains draw on natural forms of erosion, obstruction or destruction, namely of a head-shaped tree losing leaves or a face of sand being washed away by the sea (Ang et al., 2023), a paper brain on fire (Brookes et al., 2018), weeds that threaten to engulf a person or a mist descending over a character’s head (Caldwell et al., 2021). It is worth noting that, while most of these natural processes occur over a period of time, fire is an especially immediate and all-consuming destructive force that therefore risks mispresenting the progression of dementia as immediate and final, especially when the visual metaphor involves fire consuming a material that is highly combustible (e.g. Brookes et al., 2018). Nonetheless, regardless of time frame, in each of these metaphors the person with dementia is either eroded, entrapped or eradicated by the progression of the syndrome. When contextualized within the privileging of the brain and cognitive capacities in human identity and worth (Post, 2000a, 2000b; Vidal, 2009), the deterioration of the brain likely implicitly entails the deterioration of the person themselves.

The loss of self that is therefore implied by these natural metaphors is arguably made more explicit in other loss-oriented visual metaphors, whereby parts of a person’s face (particularly the area where the brain is located) are faded out or even missing entirely. Notably, people with dementia (and sometimes their brains) are objectivized as incomplete jigsaw puzzles, with a jigsaw piece from the area where the brain is located either being removed, missing or out of place (Ang et al., 2023; Harvey and Brookes, 2019). Elsewhere, a blue wash progressively covers the head of the character with dementia (Caldwell et al., 2021), or areas of the person’s face (which always include the area where the brain is located) fade into scenarios of disasters associated with memory loss (Brookes et al., 2021). In each case, aspects of dementia – namely, memory loss and confusion – are visualized through making the person physically incomplete, which again suggests that a loss of self accompanies cognitive decline with dementia. Taking this a step further, before critiquing such a discourse, the artist Walrath illustrates their character with dementia as missing their head entirely (Kovan and Soled, 2023). Whether metaphorical or literal, then, dementia and particularly negative associated aspects (such as memory loss, distress and the related discourse of loss of self) are recurringly foregrounded at the expense of the individual experiencing dementia. Relatedly, such individuals are frequently depicted as being incomplete, whether through disembodied visuals of hands/heads or by being shown with parts of themselves missing.

#### Othering people living with dementia

The means of visually representing dementia described in the previous section arguably serve to establish social distance between represented participants with dementia and others (i.e. viewers and other represented participants) through depictions that impersonalize the individual with the syndrome and focus instead on their symptoms and other facets of dementia. Objectifying people with dementia through close-up shots of body parts is a key example of how symbolic distance is established. However, when individuals and/or their faces are shown in full, then distance can also be conveyed through other semiotic choices regarding participant positioning and attributed characteristics, which is the focus of this section.

Encouraging a sense of social distance, people represented as having dementia often do not look at the camera or at other represented participants, instead either looking down, looking vacantly elsewhere, or having their eyes closed (Ang et al., 2023; Brookes et al., 2018, 2021; Harvey and Brookes, 2019; Kovan and Soled, 2023; Latimer, 2018). This lack of eye contact can signal social disconnect by impersonally ‘offering’ the represented participants with dementia to viewers to observe rather than connect with, much like ‘specimens in a display case’ (Kress and Van Leeuwen, 2006: 119). Many images can also distance people with dementia from viewers through the use of angles of interaction, which either attribute greater power to viewers (i.e. a high angle) or separate the planes of existence (i.e. oblique and non-frontal angles) (Ang et al., 2023; Brookes et al., 2018; Harvey and Brookes, 2019). Similarly, images may show supporters/carers as positioned higher up in an image than someone with dementia, thereby casting those supporters/carers in a position of power (Harvey and Brookes, 2019). Gaze and angle, then, can act to separate the worlds of viewers from represented participants with dementia through subtly indicating both physical and social distance.

Objectification and infantilization through visual arrangements of social actors or associated dress may also be used to delegitimize people living with dementia. In certain advertisements for surveillance devices, people with dementia are visually placed alongside pets, children/babies and possessions, which implicitly positions them as similar objects to be tracked rather than as human users of the advertised technology (Vermeer et al., 2022). Equally, picturebooks can infantilize adults with dementia by depicting them as dressing in a non-conformist manner, which is positioned as acceptable for children but not adults (Caldwell et al., 2021). In this way, people with dementia risk being presented as non-adults, implicitly delegitimizing their decision-making and agency.

Moreover, depersonalized settings, passive poses and dull colour palettes risk draining social status, personality and life from people with dementia. Visual settings tend to be impersonal, and often stark, with nothing to signal the individual’s personality or life, aside from perhaps the occasional wedding ring (Brookes et al., 2018, 2021; Harvey and Brookes, 2019). While generic settings are typical of stock images (Machin and Van Leeuwen, 2007), which are a key genre for many papers included in this review, when used in combination, this semiotic choice may further depersonalize individuals by denying them any sense of a distinct identity (again furthering the positioning of someone as a ‘specimen’ of people with dementia above being a unique person).

Passivizing depictions frequently show individuals with dementia as immobile (often seated alone) and with vacant, unchanging facial expressions (Ang et al., 2023; Brookes et al., 2018, 2021; Caldwell et al., 2021; Harvey and Brookes, 2019; Kovan and Soled, 2023; Latimer, 2018). Such visual positioning reinforces the traditional silencing and marginalization of people living with dementia by implying that, when people are diagnosed with dementia, they lose their agency and ability to interact with the world, despite evidence to the contrary (Bartlett and O’Connor, 2010; Kontos, 2003, 2004, 2006; Kontos et al., 2017; Sabat, 2018). Chronological multi-image narratives emphasize this distancing of people living with dementia by showing characters change from being presented as active and in close proximity to loved ones before their diagnosis, to then becoming inactive and uncommunicative, with greater physical and social distance from the other characters conveyed by either facing away or being at opposite ends of the image (Caldwell et al., 2021). Such visual depictions risk exaggerating the progression of dementia, alongside situating individuals not as *living* with the condition but as lifeless, and as lacking in individual identity.

This passive and vacant body language can be combined with a muted colour palette – namely of white, greys, blues and beiges – to implicitly position people with dementia as the ‘living dead’ (Brookes et al., 2021; Harvey and Brookes, 2019). Such hues present a lower visual modality regarding colour saturation and colour range than the naturalistic standards by which viewers are encouraged to judge reality (Kress and Van Leeuwen, 2006). They are also situated primarily at the blue end of the red–blue hue continuum, which is associated with ‘cold, calm, distance, and backgrounding’ (Kress and Van Leeuwen, 2002: 357). As such, these colours have the potential to evoke ghostliness and suffering (e.g. note the connotations of the phrases ‘grey world’, ‘white as a ghost’, ‘beige personality and ‘feeling blue’) and, when combined with other semiotic choices such as vacant gazes, this kind of colour palette can therefore contribute to positioning people living with dementia as ‘listless, vacant and fading souls’, distinct from the ‘world of the living’ (Brookes et al., 2021: 256–257).

Finally, it has been noted that certain social groups are overrepresented, while others are backgrounded or excluded, which may potentially contribute to stereotypes of people diagnosed with and otherwise affected by dementia. Notably, younger women are overrepresented in the carer role, while older adults (especially women) are depicted as having dementia (Ang et al., 2023; Brookes et al., 2021; Caldwell et al., 2021; Venkatesan and Kasthuri, 2018). Similarly, white faces are overrepresented (Ang et al., 2023; Vermeer et al., 2022). While not always explicitly linked to stigma by the publications, we would argue that such patterns present a skewed stereotype of people affected by dementia, which ignores the diversity of this group and potentially intersects with other forms of stigma (here, sexism, ageism and racism). However, more detailed analysis by the studies would be required to examine this intersectionality further. Dementia stigma is most often discussed in relation to ageism by studies since, although young onset dementia (whereby symptoms occur before the age of 65) accounts for up to 9% of diagnoses worldwide (World Health Organization, 2023), visual cultural markers of older age are consistently used (such as wrinkles and grey hair) to visually conflate dementia with the later life stages (Ang et al., 2023; Brookes et al., 2018; Caldwell et al., 2021). As the above sections demonstrate, this ‘ageing–dementia relation’ is often presented negatively, likely reflecting that dementia has become symbolic of society’s worst fears about ‘ageing unsuccessfully’ (Latimer, 2018).

Evidently, people with dementia may be impersonalized not only through the foregrounding of dementia, but also through consistent semiotic choices which visually frame people with dementia as socially and physically different. This can be achieved through visual framing techniques (angle, gaze, colour, setting) and attributed characteristics (notably, subtly positioning people with dementia as the living dead). Moreover, stereotypes regarding who is represented in these studies potentially indicate the intersectionality of dementia stigma with other stigmas, particularly ageism.

### Challenging dementia stigma?

There is much less research on ways in which visual representations might challenge dementia stigma, with Vermeer et al. (2022: 416) actually ‘questioning if non-stigmatising representations are fathomable or culturally possible’. There appear to be four aspects of visual depictions that authors indicate may help to challenge, rather than contribute to, dementia stigma. These include: (1) emphasizing social connection rather than distance; (2) replacing visual metaphors of loss with a metaphor of transformation; (3) visually exploring the perspective of someone with dementia; and (4) reinterpreting supposedly ‘stigmatizing’ images. We will explore each of these in turn.

The first and most frequently discussed visual trope which might challenge dementia stigma is that of showing a person with the syndrome as someone to socially connect with, not to be distanced from. Many of the visual features examined are therefore opposite to those discussed above. Firstly, authors determine that stigma can be challenged through showing the person’s face within the frame (and likely most of their body too) and foregrounding the individual as having an identity, agency and a socially equal status to viewers or others in the image. This can in part be achieved through showing people living with dementia making eye contact with viewers or other represented participants in the image, and through using a frontal angle to better align viewers with the person’s world (Ang et al., 2023; Harvey and Brookes, 2019). If using the direct form of gaze, represented participants with dementia can thus ‘demand’ a social relationship with viewers, and the nature of this relationship can be determined by the person’s facial expressions and actions (Kress and Van Leeuwen, 2006). As such, authors also advocate for portraying someone with dementia as agentively participating in an activity, whether social or otherwise, and as displaying an emotion of positivity and hope, for example by laughing, smiling and visually engaging with other participants, including by showing affection (Ang et al., 2023; Harvey and Brookes, 2019; Latimer, 2018; Vermeer et al., 2022). Emphasizing social connection in this way may in turn facilitate more socially embedded notions of memory and personhood, which can better support the continued identities and interactions of people living with dementia (Brookes et al., 2021). This is exemplified by some of the picturebooks examined by Caldwell et al. (2021: 125), in which child characters are ‘the holder of memory’, often sitting on their grandparent’s lap and sharing memories or meaningful items. If the person is shown alone, Ang et al. (2023) argue that the image should connote independence, rather than loneliness.

Moreover, visual metaphors can conceptualize dementia not in terms of loss but in terms of transformation and, relatedly, be used creatively alongside non-metaphorical depictions to explore the perspective of a person living with dementia. It is worth noting that the examples for these alternative visual metaphors are all drawn from Dana Walrath’s graphic novel, *Aliceheimer’s: Alzheimer’s Through the Looking Glass*, which is about Walrath’s mother, Alice, for whom Walrath cared. This graphic novel is analysed by multiple papers included here (Kovan and Soled, 2023; Venkatesan and Ancy, 2023; Venkatesan and Kasthuri, 2018). Notably, the visual metaphor of a person with dementia (Alice) becoming ungrounded and eventually flying with dementia disrupts the traditional notion of a disappearance of self by instead depicting a transformation. More specifically, Alice is shown with her feet off the ground, and then flying in a Superman pose next to a bird above the mountains (Venkatesan and Ancy, 2023; Venkatesan and Kasthuri, 2018). Relatedly, visual metaphors can also be used to potentially make the world of someone living with dementia more accessible to others. This requires personalizing the metaphors used to the individual, as Walrath does for Alice. For instance, building on her mother’s experiences and intertextual references, Alice is presented as being in a magical world or ‘wonderland’ (linking to Alice in Wonderland) with haloes and multi-coloured stars, in which one can float around an orange teapot and have broccoli growing from one’s ears (Venkatesan and Kasthuri, 2018). Equally, illustrations can be used to humanize Alice’s ‘shifting life world in terms of space, place, and time’, for instance, by showing Alice as seeing her late husband in the branches of a maple tree, which can render her experience more relatable to viewers (Kovan and Soled, 2023: 238).

Finally, building on the ‘plurality of possible interpretations’ for any social text (Widdowson, 1998: 150), the same image or visual trope that could be interpreted as contributing to stigma can instead be interpreted in ways that complicate or challenge it. Notably, not all disembodied shots, here of people’s hands, are straightforwardly stigmatizing; as mentioned previously, images of holding/touching hands may instead signify comfort and support, and different hand sizes and textures can signal intergenerational care (Ang et al., 2023). Such themes align more with the visual trope of emphasizing social connection, rather than distance, and could thus be argued as having the potential to challenge stigma. Similarly, Latimer (2018) convincingly argues that the typical image of someone appearing socially disconnected can instead be reinterpreted either as a critique of stigma itself, namely the marginalization of people living with dementia, or, more radically, as challenging contemporary ideologies of what it means to be a person by showing just one of *many* ways of being human.

## Discussion

This article reviews research exploring the relationship between stigma and visual representations of dementia and people with dementia. Our review indicates that this topic has only recently gained interest, with all research on it being published from 2018 onwards. This research is overwhelmingly qualitative in its orientation, and examines a range of visual data types, including books, newspapers, websites, print advertisements, public health promotion posters and YouTube videos. From our synthesis, it is worth noting that data types with multiple or moving images (namely videos, graphic novels and picturebooks) seem to have much greater potential for more complex visual representations of people with dementia. Notably, chronological stories can be told (e.g. through following someone over time with dementia) and different discourses can be used to challenge audience expectations (e.g. Walrath’s illustration of a disappearing head image is confronted and replaced by the visual metaphor of transformation and illustrations that explore Alice’s perspective). If an image is viewed as ‘an act of perception and construction which frames a world’ (Featherstone and Wernick, 1995: 4), it is indeed logical to suppose that multiple or moving images can provide a more comprehensive and thus potentially more complex worldview. The choice of mode, and medium, is therefore an important consideration for future research into dementia-related stigma and for anti-stigma initiatives.

Theorizations of stigma do not seem to explicitly guide any of the studies; indeed, 70 percent of included papers do not even provide a definition of stigma, reinforcing existing findings that dementia stigma is under-theorized (Nguyen and Li, 2020; Werner, 2014). However, the general principle that underlies the majority of stigma theories – that of establishing or challenging distance between an in-group (‘us’) and out-group (‘them’) – does seem to inform many of the studies reviewed. While it is of course not necessary to draw on any stigma theories when analysing visual communication of dementia, we would argue that such theories do have the potential to be useful to visual researchers, including regarding interpretation and dissemination. Notably, as we aim to evidence below, interpreting visual communication in relation to theories of stigma and stigma reduction can help to situate visual texts within broader social practices, and advocate for greater attention to be paid to visual communication in the global efforts to address dementia stigma. Drawing on stigma theories when *producing* visual texts may also help to inform discussions of the implications of particular semiotic choices (such as gaze) and who is involved in the text production itself (notably, are people with or otherwise affected by dementia (co)producers?). Deeper engagement with theorization around stigma could thus unlock, to researchers in visual communication and text designers alike, the resources for better understanding and communicating more effectively about different types of stigma, and the particular impacts that these can have on those living with a dementia diagnosis. It is our hope, then, that considering the relationships between the fields of visual analysis and stigma can inform research and advocacy efforts moving forward.

Visual features that have the potential to contribute to dementia-related stigma appear to map especially well onto Link and Phelan’s (2001, 2006) modified labelling theory, which envisions stigma as the co-occurrence of five elements: labelling certain human differences as meaningful, negative stereotyping, a separation of ‘us’ from ‘them’, status loss and discrimination, and the exercise of power. People with dementia are consistently visually labelled in relation to dementia through foregrounding particular symptoms or other aspects of dementia (such as loss and suffering) above the individual themselves. These labels are in turn tied to negative and reductive stereotypes of people with dementia, including that such individuals undergo drastic change and even a loss of self, and that people with dementia are therefore incomplete, disengaged, lifeless and dangerous to themselves and others. Such stereotypes contribute to a separation of people with dementia (‘them’) from viewers (‘us’), since they imply that people with dementia are fundamentally different, and perhaps even inferior, to people without dementia. Specific visual techniques combine with such stereotypes to establish symbolic distance between people with dementia and viewers and/or other represented participants without dementia. These techniques tend to involve the use of angles that separate the planes of existence (i.e. not frontal or eye level), impersonal settings and shots that do not provide eye contact, whether through indirect gaze or close-ups that do not show the face. Higher angles may also sometimes be used to disempower people with dementia relative to other represented participants (such as carers) or viewers, which reflects the fifth essential component of stigma: the exercise of social, cultural, political and economic power against the stigmatized group (Link and Phelan, 2001, 2006). Occasionally, there are also visual indicators of status loss and discrimination associated with dementia, which is perhaps most evident in the surveillance advertisements that place people with dementia alongside pets, babies and possessions, implicitly positioning such individuals as items to be tracked rather than potential users of the technology (Vermeer et al., 2022).

It is also worth briefly considering why the particular visual choices that are associated with dementia may contribute to stigma. For this, we draw on Jones et al.’s (1984) multiple dimensions of stigma, which suggest that stigma is worse if a condition is: (1) visible (rather than concealable); (2) at an advanced stage or if it will become more salient and debilitating over time; (3) disrupts interpersonal relationships; (4) is unattractive; (5) if the person is responsible for the attribute’s origin; and (6) if the condition is associated with threat/danger, whether physically or existentially (e.g. through an association with death). With the exception of responsibility for dementia’s origin, all of these aspects arguably inform the visual representations of people with dementia in this review. Despite lacking in physical markers, aspects of dementia are clearly made visible for viewers, including by representing signs (such as memory loss), associated attributes (vacant or pained expressions) and neurodegeneration (e.g. through visual metaphors of neurodegenerative loss). These are presented as the visible markers of difference with dementia, which can then be used to label and negatively stereotype certain bodies relative to others (Kessler, 2022), especially when interpreted in relation to the remaining dimensions. Firstly, there is an overall emphasis on more advanced dementia, with people being positioned as vacant, ‘stagnant’ (Kovan and Soled, 2023: 235) and deteriorating (e.g. through loss-oriented ‘before’ and ‘after’ images or visual metaphors). Relatedly, dementia is visually associated with markers of older age (e.g. wrinkles) and death (e.g. immobile positions, ghostly colour palettes), both of which tend to be deemed aesthetically and existentially threatening to a society that ‘resist[s] the thought of bodily decline, deep old age and our eventual death’ (Featherstone and Wernick, 1995: 2). While degeneration and death offer an implicit threat, peril is more explicitly visually associated with dementia through foregrounding dangerous situations and, in one instance, the economic threat of people with dementia. Evidently, the visual aspects of dementia identified as potentially contributing to dementia stigma align closely with theorized contributing factors for increased stigma.

Equally, the few features identified as having the potential to help challenge dementia stigma can also be usefully interpreted in relation to the existing anti-stigma literature. Bacsu et al.’s (2022) scoping review of dementia stigma reduction interventions is particularly pertinent here. The authors draw on Corrigan and Penn’s (1999) stigma-reduction framework – which features protesting harmful representations, education and contact with the stigmatized group – to evaluate a range of interventions in a dementia context and, although visual arts programmes (creating plays, artwork, etc.) are included, the potential for the visual modes themselves to help challenge stigma is not considered. Yet, the visual representations reviewed here also show the potential for protest, education and contact, and they align well with Bacsu et al.’s (2022) key components of dementia specific stigma reduction interventions. First, the visual mode tends to ensure that the information is brief and accessible, a key proposed feature of education interventions (although, as Robson, 2022, argues, since visual artefacts mean different things to different people, there is potential for messiness and confusion). Secondly, the visual representations focus on positives rather than negatives, position dementia as more than memory loss and demonstrate that it is possible to lead an active life with dementia (Bacsu et al., 2022), for instance, through showing people enjoying participating in activities, social or otherwise. Relatedly, social connection is encouraged between people with dementia and viewers/other represented participants (e.g. through direct gaze, frontal/eye-level angles and showing social interaction), which not only challenges existing tropes of disengagement and distance, but also symbolically increases *contact* with people with dementia. The same can be said of visual representations that take the perspective of the person with dementia. Meanwhile, alternative metaphors, here of dementia as transformation, can help to protest more dominant ones (i.e. of dementia as loss) and create ‘opportunities for action and “escape”’ from the dominant narrative (Bennett, 2023: 49). What appears to be lacking, though, is the creative input and self-representation of people living with dementia, which is central to so many other stigma reduction initiatives and requires much greater consideration in the context of visual representations of dementia (Bacsu et al., 2022; Bennett, 2023; Corrigan and Penn, 1999; Kessler, 2022). While research has begun to recognize the value of exploring the self-representations of people with dementia (e.g. Lim et al., 2021; Ludwin and Capstick, 2015), the same cannot (yet) be said for the types of mainstream media analysed in the studies included here.

When considering ways of challenging stigma, it is important to examine the reception of such attempts. Indeed, re-interpreting potentially stigmatizing visual representations in ways that instead emphasize connection, compassion and acceptance is the final feature noted in this review. However, as Clair et al. (2016) highlight, the perceived credibility of new (or re-interpreted) visual representations and their wider discourses must be considered. Complicating anti-stigma efforts, images of loss can still be well received by people with experience of dementia as helping to explain the complex reality of neurodegeneration (Ang et al., 2023; Putland, 2022). Meanwhile, an overemphasis on positivity with dementia can also be criticized for ignoring the suffering associated with dementia and excluding more vulnerable people with advanced dementia (Bartlett et al., 2017; McParland et al., 2017). A binary between overly negative and overly positive visual representations must be avoided then (McParland et al., 2017) and more inclusive and nuanced alternatives need to be explored further as part of advocacy efforts.

Inevitably, this review cannot provide an entirely comprehensive picture of research on visual representations of dementia and stigma. Some limitations are procedural; our search strategy and inclusion criteria may have caused us to miss publications that would provide additional or opposing insights to those presented here. Notably, no ‘grey literature’ (e.g. doctoral theses, conference contributions, posters) was included, nor were studies published in languages other than English, which likely contributes to the Western (especially Anglo) bias of our data. As reviewers, we were also limited by what the authors of included publications chose to discuss and the visual examples that they included since we did not have access to their whole datasets. We may therefore have missed other potentially important patterns and discrepancies across the datasets themselves. Equally, in focusing here on visual representations, we ignore the interactions between different modes of communication, for instance of visual modes with text, speech or music. Such a focus is recommended as a direction for future research, as is a consideration of more diverse datasets and cultural contexts. A final issue to consider, which might be regarded as a limitation, pertains to the fact that the authors of this review were also authors of three of the publications we have reviewed. This, in our view, does not pose any serious limitation since our review of the publications has aimed to be primarily descriptive (rather than critical). Nevertheless, given the small number of multimodal studies on dementia stigma overall, these three studies represent a considerable portion of the overall body of work we have reviewed. Of course, as further work is carried out at the interface of multimodality, dementia and stigma (as we hope it will be), then the prospect of this issue arising in the future shrinks in size, both for us and others working in the area.

Reflecting on some of the main features and limitations of the research reviewed here, future studies on visual representations and dementia stigma should aim to more comprehensively engage with stigma as a theoretical construct, attending to both how features might contribute to and/or challenge dementia stigma. The latter effort seems to be in the especially early stages, and a consideration of anti-stigma visualizations would benefit greatly from critical engagement with the nuances and ethics of such an endeavour, which is starting to be grappled with in a broad sense but not in relation to visual communication (e.g. Fletcher, 2021). As part of this, centring the perspectives of people living with and otherwise affected by dementia for both researching and creating visual representations is essential. So, too, is a focus on the intersectionality of stigma, which could greatly improve understandings of more diverse experiences of dementia and how multiple stigmas may interact (e.g. see Hulko, 2009; King, 2022; O’Connor et al., 2010).

## Data Availability

Data sharing is not applicable to this article as no datasets were generated or analysed during the current study.

## Notes

1. Note that, to keep the scope of our review as broad as possible, we did not stipulate that the included studies followed any particular model or theorization of stigma. For the same reason, we have not aligned ourselves in this article with any particular theorization of stigma (for comprehensive reviews of dementia stigma, see Nguyen and Li, 2020; Pescosolido and Martin, 2015; Werner, 2014).
